# Caffeine Consumption Habits of New Zealand Tertiary Students

**DOI:** 10.3390/nu13051493

**Published:** 2021-04-28

**Authors:** Saskia Stachyshyn, Ajmol Ali, Carol Wham, Tayla Knightbridge-Eager, Kay Rutherfurd-Markwick

**Affiliations:** 1 School of Sport, Exercise and Nutrition, Massey University, Auckland 0632, New Zealand; saskiastash@hotmail.com (S.S.); C.A.Wham@massey.ac.nz (C.W.); T.Eager@massey.ac.nz (T.K.-E.); 2 Centre for Metabolic Health Research, Massey University, Auckland 0632, New Zealand; K.J.Rutherfurd@massey.ac.nz; 3 School of Health Sciences, Massey University, Auckland 0632, New Zealand

**Keywords:** side effects, safe limit, coffee, energy drink, ready to drink, tea

## Abstract

Adverse effects associated with excessive caffeine consumption combined with increasing numbers and availability of caffeine-containing products are causes for concern. Tertiary students may be at increased risk of consuming excessive amounts of caffeine due to seeking caffeinated products with well-known wakefulness effects and cognitive benefits. This study explored caffeine consumption habits of New Zealand tertiary students (317; ≥16-years) using a previously validated caffeine consumption habits (CaffCo) questionnaire. Most (99.1%) regularly consumed caffeinated products, especially chocolate, coffee and tea, with coffee, tea and energy drinks contributing most to total caffeine intake. Median estimated caffeine intake was 146.73 mg·day^−1^, or 2.25 mg·kgbw^−1^·day^−1^. Maximum and minimum intakes were 1988.14 mg·day^−1^ (23.51 mg·kgbw^−1^·day^−1^) and 0.07 mg·day^−1^ (0.02 mg·kgbw^−1^·day^−1^), respectively. One-third (34.4%) of caffeine consumers ingested caffeine above the adverse effect level (3 mg·kgbw^−1^·day^−1^) and 14.3% above the safe limit (400 mg·day^−1^). Most caffeine consumers (84.7%), reported experiencing at least one ‘adverse symptom’ post-caffeine consumption, of which 25.7% reported effects leading to distress or negatively impacting their life. Experiencing ‘adverse symptoms’ did not, however, curtail consumption in the majority of symptomatic participants (~77%). Public health initiatives directed at tertiary students may be important to reduce potential caffeine-related harm.

## 1. Introduction

Caffeine is one of the world’s most widely used mood and behaviour altering drugs [1]. An estimated 80% of people worldwide regularly consume caffeine [2]. It has been estimated that approximately 73% of New Zealanders consume caffeine daily, with an average daily caffeine intake of 3.6 mg·kgbw^−1^ [3]. This estimate is higher than the US (2.4 mg·kgbw^−1^·day^−1^) [4,5], lower than Denmark (6.7 mg·kgbw^−1^) [4] and similar to Argentina (4.3 mg·kgbw^−1^) [6], the United Kingdom (UK; 4.1 mg·kgbw^−1^) [4] and Japan (3.7 mg·kgbw^−1^) [7].

The caffeine content of different products can vary greatly. Products which naturally contain caffeine such as coffee, tea and chocolate may differ in caffeine content due to differences in growing conditions, and processing and brewing techniques [8,9,10]. While the caffeine content of coffee, tea and chocolate is not regulated, the Food Standards Australia New Zealand Code stipulates that the caffeine content of kola-flavoured beverages must not exceed 145 mg·L^−1^, while energy drinks (‘formulated caffeinated beverages’) must contain between 145 and 320 mg·L^−1^ of caffeine [11]. Dietary supplements, sports supplements and caffeine tablets are exempt from caffeine-related regulations [12].

From the limited studies assessing caffeine intake in New Zealand (NZ) [13,14,15], it appears that when energy drinks are excluded, coffee, tea and soft drinks contribute the most to the baseline caffeine intakes of New Zealanders; this is similar to consumption patterns observed in Australia [16] and the US [5].

Based on the ‘adverse effect level’ of 3 mg·kgbw^−1^·day^−1^, an assessment of dietary caffeine exposure, excluding energy drinks, revealed that a quarter (26%) of NZ adults (≥15 years) may be at risk of the adverse effects of caffeine [17]. However, as this excludes energy drinks the true prevalence of at-risk individuals may be greater. Furthermore, there are no international guidelines for a ‘safe limit’ of daily caffeine intake although, levels up to 400 mg·day^−1^ are generally regarded as ‘safe’ for non-pregnant adults by multiple agencies and reviews [2,18,19,20,21].

Caffeine is well known for its positive effects, such as increasing alertness and combating fatigue [22,23,24]. Similarly, Cohen et al. [25] reported that tertiary students perceived that they were significantly more alert, awake, clear-minded and able to concentrate after ingesting low doses of caffeine; such effects are desirable for academic work. Alternatively, excessive caffeine consumption can cause negative symptoms ranging from anxiety, nausea, palpitations, upset stomach, headaches and sleeplessness through to respiratory problems, liver and heart damage, seizures and death [26].

Caffeine response varies between individuals due to many factors, including dosage, genetics, tolerance [27] and the activity of the caffeine-metabolising enzyme cytochrome P450 1A2, the activity of which differs between individuals [28,29,30,31,32,33,34,35]. Thus, the dose of caffeine which causes negative effects varies between individuals.

Habitual consumption of caffeine can result in the development of a physiological tolerance [36,37]. When a habitual caffeine consumer abruptly reduces or stops consuming caffeine, they are likely to experience symptoms of withdrawal ranging from a headache to dysphoric mood [36,38]. In NZ there is no official reporting of caffeine-related health incidents. However, such incidents have escalated worldwide. Considering energy drink-related incidents alone, emergency department visits in the USA doubled (10,068 to 20,783) between 2007 and 2011 [39] and of the 297 incidents reported to Australia’s poisons centre between 2004 and 2010, 43% resulted in hospitalisation [40]. Furthermore, as this data pertains to energy drink-related harm only, it is likely an underestimation of the true impact of caffeine exposure on health systems.

Since the benefits and risks of caffeine consumption are dose dependent, potential risks can only be determined once current consumption patterns have been assessed. Tertiary students may be at an increased risk of consuming excessive amounts of caffeine due to its effect on boosting wakefulness and cognition. Therefore, this study aimed to quantify caffeine intake and to explore the caffeine related experiences (i.e., symptoms post consumption, dependence and withdrawal) of NZ tertiary students.

## 2. Materials and Methods

### 2.1. Study Design

As a part of a larger cross-sectional study “Caffeine Consumption Habits, Motivations, and Experiences of New Zealand Tertiary Students” [41], this investigation utilised a validated caffeine consumption habits (CaffCo) questionnaire [42] to explore caffeine intake from a range of caffeinated products from a convenience sample of tertiary students in New Zealand.

### 2.2. Participants

Three-hundred-and-seventeen tertiary students (*n *= 169 females) participated in this study. Participants were aged ≥16 years and were not required to consume caffeinated products to participate in this study.

Ethical approval was gained from the Massey University Human Ethics Committee: Southern A (SOA 15/76) before the commencement of data collection. Participants were not able to remain anonymous to the researchers due to the in-person nature of the data collection, however, all data was anonymised by assigning a unique identifier (six-digit numerical figure) to each participant.

### 2.3. Experimental Procedures

An online survey was used to conduct this study between June–August 2016. Participants were recruited via media releases in newspapers, poster advertisements, in person at the time/place of data collection and by word of mouth.

Persons who volunteered to participate were able to complete the questionnaire at various points of data collection (Massey University Albany, Massey University Palmerston North, and University of Auckland City campus) or at a location of their choice (by following a link to the questionnaire).

Screening questions were incorporated into the online questionnaire, which was administered using online survey software [43]. Inclusion criteria were being aged ≥15 years-old and being a tertiary student (i.e., currently enrolled in either part-time or full-time study at a higher education facility). Persons aged 14 years and under were excluded to allow the results obtained from this research to be aligned with the data from the 2008/2009 NZ Adult Nutrition Survey University of Otago and Ministry of Health [13].

### 2.4. Caffeine Consumption Habits (CaffCo) Questionnaire

Designed to examine caffeine habits, consumption patterns, positive and negative effects or experiences, and influences, over a range of caffeinated products, the caffeine consumption habits (CaffCo) questionnaire is a validated online questionnaire which was developed and pilot-tested in 2015 [42]. The CaffCo showed good validity and reliability in NZ adults aged ≥ 15 years to identify patterns of caffeine consumption across a range of caffeinated products [42].

In this sub-study, the Food Frequency Questionnaire (FFQ) component of CaffCo was utilised to gather details on serving size and the frequency of consumption of 34 caffeine-containing products, including tea, coffee, chocolate, kola drinks, energy drinks, caffeinated ready-to-drink (RTD), sports supplements and caffeine tablets. Further data collected from CaffCo included information on self-reported caffeine dependence, caffeine withdrawal symptoms and socio-demographic and anthropometric data. Body mass index (BMI) was calculated using self-reported height and weight data.

### 2.5. Statistical Analysis

The CaffCo questionnaire data was first exported from Qualtrics into Microsoft Excel where it was screened for missing information. Data was then exported into a statistics package (IBM SPSS Statistics for Windows, Version 25.0. Armonk, NY, USA) for statistical analysis. Participants were categorised by gender and age groups (16–18 years, 19–30 years, 31–50 years and ≥51 years).

The estimated daily caffeine consumption was calculated for every caffeine-consuming participant by combining the caffeine concentration data for various caffeine-containing products [44] with the consumption frequency data from the CaffCo questionnaire. Different consumption frequencies were assigned a factor according to their relationship to daily consumption (e.g., if the consumption frequency was once a week, the factor would be 1/7 = 0.143). If the consumption frequency included a range, the middle value would be used (e.g., 2–3 times a day would be a factor of 2.5).

Descriptive statistics were used to summarise participants’ socio-demographic and anthropometric characteristics. Scale variables were tested for normality by carrying out Kolmogorov-Smirnov and Shapiro-Wilk tests. All scale data were non-parametric and therefore reported as median and interquartile range (IQR). Categorical data was reported as frequency and percentage.

The contribution of each caffeine source to the total daily caffeine consumption was calculated by aggregating the caffeine consumption of all participants from that source and expressing this as a percentage of the total caffeine consumed by all participants.

Contingency tables were used to compare percentage consumption of the caffeine sources according to different demographic and participant characteristic groups. Since all scale data was non-parametric, Mann-Whitney U-tests, Kruskal-Wallis tests and Kendall’s Tau correlations were used. *p *< 0.05 was indicative of statistical significance for all tests.

For 2 × 2 contingency tables, if all expected counts were 10 or greater, the Pearson’s chi-squared test for independence was used. If any of the expected counts were less than 10 but greater than or equal to 5, the Yates continuity correction was applied. If any of the expected counts were less than 5, the Fisher exact test was used [45]. For contingency tables larger than 2 × 2, the Pearson’s chi-square test of independence was used on the condition that “no more than 20% of the expected counts are less than 5 and all individual expected counts are 1 or greater” [46]. If these conditions were not met, the Fisher’s exact test was used. If contingency tables larger than 2 × 2 reached significance, post hoc testing was carried using multiple 2 × 2 contingency tables and the stepwise Holm-Bonferroni method. For contingency tables which showed significance, the odds ratio was also calculated to show the practicality of the significance.

If the Kruskal-Wallis tests showed significance, post hoc testing using multiple Mann-Whitney U-tests and the stepwise Holm-Bonferroni method was carried out. If significance was reached for any Mann-Whitney U-test, the effect size (r) was calculated in order to show practical significance, using the formula; r = z/√N [47]. A value of 0.1 signifies a ‘small’ effect size, 0.3 signifies a ‘medium’ effect size and 0.5 signifies a ‘large’ effect size [48].

For Kendall’s Tau correlations, Cohen’s standard was used to determine the strength of the relationship. Correlations between 0.10 and 0.29 signified a small association; correlations between 0.30 and 0.49 signified a medium association; and correlations of 0.50 and higher signified a large association [25].

## 3. Results

### 3.1. Participants

A total of 318 participants met the inclusion criteria for this study and completed the CaffCo questionnaire. Lacking sufficient statistical power for analysis, one further participant was removed from the dataset as they identified their gender as ‘other’. Thus, the final dataset consisted of 317 tertiary students, aged ≥16 years old.

Most of the participants were female (*n *= 169, 53.3%), NZ European (*n *= 150, 47.5%), non-smoking (*n *= 268, 84.5%), and aged 19–30-years-old (*n *= 236, 74.4%; Table 1). Most participants provided their height (*n *= 263) and/or weight (*n *= 281) data, from which BMI was calculated (for *n *= 263, 83%). The median BMI was 22.9 kg.m^−2^ (IQR = 20.8–25.1). The BMI of male participants (M = 23.4 kg·m^−2^) was significantly higher than that of females (M = 22.3 kg·m^−2^; U = 7137, *p *= 0.014, r = −0.15). Further participant characteristics are published elsewhere [41].

### 3.2. Caffeine Intake

Most participants (99.1%) reported regular consumption of caffeine-containing foods and beverages. The median estimated total daily caffeine intake was 146.73 mg·day^−1^. Coffee contributed the greatest amount to total daily caffeine intake (61.4%), followed by tea (14.4%), energy drinks (8%), chocolate (7.3%), kola drinks (5.3%), caffeine-containing sports supplements (2.4%), caffeinated RTD (0.8%), and caffeine tablets (0.5%).

For the participants who provided body weight data (*n *= 281) the median relative daily caffeine consumption was 2.25 mg·kgbw^−1^·day^−1^ (IQR 1.01–4.31 mg·kgbw^−1^·day^−1^). The maximum estimated daily intake by a single person was 1988.14 mg·day^−1^, or 23.51 mg·kgbw^−1^·day^−1^, and the lowest estimated intake from all sources was 0.07 mg·day^−1^ or 0.02 mg·kgbw^−1^·day^−1^; these values reflect the daily consumption of 4–5 double shot espressos (providing an estimated 945 mg caffeine) and a single cup of hot chocolate less than once a month, respectively.

There was no significant difference in estimated total daily caffeine consumption between males and females (*p *= 0.703). However, when expressed relative to body weight, daily caffeine consumption was significantly higher in females than males (U = 8289, *p *= 0.041, r = −0.123). Daily consumption of caffeine from kola drinks (U = 2046.0, *p *< 0.001, r = −0.283), energy drinks (U = 1191.0, *p *< 0.001, r = −0.360), and caffeinated RTD (U = 275.0, *p *= 0.022, r = −0.301) was higher in males than females (Table 2). There was no difference between males and females for daily consumption of caffeine from any other sources (*p *> 0.05).

### 3.3. Exceeding the Adverse Effect Level (3 mg·kgbw^−1^·day^−1^)

Over a third of caffeine consumers (*n* = 108, 34.4%) exceeded the ‘adverse effect level’ of 3 mg·kgbw^−1^·day^−1^ [17]. Smokers were 3.34 times more likely to exceed this level than non-smokers (χ^2^ (1) = 15.680, *p *< 0.001). There was an association between age group and the likelihood of exceeding 3 mg·kgbw^−1^·day^−1^ of caffeine (*p *= 0.002), with post hoc testing revealing that compared to the 16–18-year-old age group, the 31–50-year-old and 19–30-year-old age groups were 5.88 times (*p *= 0.002) and 2.94 times (*p *= 0.006) more likely to exceed the ‘adverse effect level’, respectively.

There was an association between consuming caffeine tablets, coffee, alcoholic RTD and tea and the likelihood of exceeding the ‘adverse effect level’. Consumers were 9.38 times (*p *= 0.001), 8.38 times (*p *< 0.001), 1.91 times (*p *= 0.026) and 1.88 times (*p *= 0.023) more likely to exceed 3 mg·kgbw^−1^·day^−1^ than those who did not consume caffeine tablets, coffee, RTD and tea, respectively.

Of participants who reported adverse symptoms, the median daily caffeine intake (mg‧kgbw^−1^‧day) was greatest in participants who experienced excitement (2.93 mg‧kgbw^−1^‧day), restlessness (2.86 mg‧kgbw^−1^‧day), inability to sleep (2.84 mg‧kgbw^−1^‧day), twitches (2.82 mg‧kgbw^−1^‧day) and a hot or red face (2.69 mg‧kgbw^−1^‧day). The median daily caffeine intake did not exceed the adverse effect level for any individual adverse symptom [41].

### 3.4. Exceeding the Suggested ‘Safe Limit’ (400 mg·day^−1^)

The suggested safe limit of 400 mg of caffeine per day was exceeded by 14.3% (*n *= 45) of caffeine consumers in this study. Participants who smoked were 3.58 times more likely to exceed this level (χ^2^ (1) = 11.694, *p *= 0.001). The consumption of coffee and RTD were also associated with the likelihood of participants exceeding 400 mg·day^−1^ of caffeine. Coffee consumers were 16.29 times more likely to exceed the safe limit than those who do not consume coffee (χ^2^ (1) = 13.752, *p* < 0.001) and alcoholic RTD consumers were 2.26 times more likely to exceed this limit than those who do not (χ^2^ (1) = 5.303, *p *= 0.025). No other sources of caffeine were associated with caffeine intake exceeding the daily caffeine ‘safe limit’ (*p *> 0.05).

### 3.5. Perceived ‘Adverse Symptoms’ Post Caffeine Consumption

**Figure 1:**
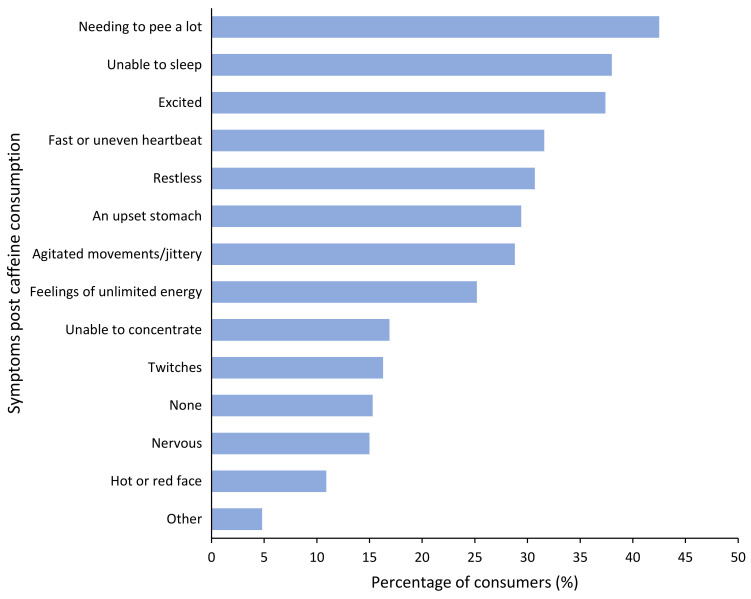
Perceived ‘adverse symptoms’ post consumption of caffeine (*n* = 314).

Of the total sample of caffeine consumers, 84.7% (*n *= 265) reported at least one ‘adverse symptom’ post-caffeine consumption. The most common reported ‘adverse symptoms’ after caffeine consumption were “needing to pee a lot” (42.5% of consumers), “unable to sleep” (38%) and feeling “excited” (37.4%; Figure 1). Of those who reported at least one ‘adverse symptom’, one-in-four (*n *= 68, 25.7%) reported that these effects had a negative impact on their social life, work life or caused some kind of distress. However, despite experiencing ‘adverse symptoms’ after consuming energy drinks and coffee, these products were still regularly consumed by 77.3% and 76.9% of symptomatic participants, respectively. For all caffeine sources, regular consumers were significantly more likely to report at least one ‘adverse symptom’ post-consumption than non-consumers (*p *< 0.05).

### 3.6. Caffeine Dependence

Of the total sample of caffeine consumers, 64.2% (*n *= 201) reported experiencing dependence on at least one caffeine source. Caffeine sources which had the highest reported consumer dependence were coffee (59.3%) and energy drinks (32.8%; Table 3).

### 3.7. Withdrawal Symptoms

Over half of the caffeine consumers (*n *= 165, 52.5%) reported no withdrawal symptoms shortly after stopping consumption of caffeine; the remaining caffeine consumers (*n *= 152; 48.4%) experienced at least one withdrawal symptom, of which over a quarter (*n *= 42, 27.6%) experienced more than one symptom. The most common caffeine withdrawal symptom was “marked tiredness or drowsiness” (31.2%), followed by “difficulty concentrating” (26.8%), “mood changes” (22.9%), “headaches” (21.3%) and “flu-like symptoms” (7.0%). These withdrawal symptoms negatively impacted on social life or work life, or caused some kind of distress in almost half (*n *= 68; 44.7%) of the participants who reported them.

## 4. Discussion

This study sought to examine the caffeine consumption habits and experiences of NZ tertiary students to determine the extent of excessive caffeine consumption in this group, which could lead to caffeine-related health-risks.

The majority (99.1%) of tertiary students who participated reported regularly consuming at least one source of caffeine in their diet. This is higher than the estimated 88% of NZ adults (≥15 years-old) previously reported in the NZ Adult Nutrition Survey 2008/09 (NZ ANS08/09) [17] and higher than rates of caffeine consumption in the USA (89%) [49]. Other studies which have examined caffeine consumption in tertiary students show the majority (87.8–98%) regularly consume caffeine from one or more sources [50,51,52,53,54,55]. It is possible that the slightly higher proportion of caffeine consumers reported in the current study is due to the study recruitment procedures. The advertisement posters (displaying a cup of coffee) may have been attention-grabbing and more attractive to caffeine consumers than non-caffeine consumers.

Chocolate, coffee and tea were the caffeine sources consumed with the highest prevalence (81.7%, 76.3% and 71.9% respectively). However, chocolate contributed only 7.3% of the total daily caffeine intake due to its low caffeine content and low total intake. Coffee was the largest contributor to estimated daily caffeine intake (61.4%), followed by tea (14.4%) and energy drinks (8%). A study exploring trends in intake and sources of caffeine in the diets of adults (≥19 years-old) in the USA also established coffee (64%) and tea (16%) as the greatest contributors to caffeine intake, however in this study soft drinks and energy drinks provided 18% and <1%, respectively [49].

Research in other countries also suggests high coffee consumption is common among tertiary students. Similar to the current study, coffee (50.8%), tea (34.8%), energy drinks (9.2%) and kola (4.7%) were the beverages which contributed the most towards total caffeine by Dutch tertiary students over a 24 h period [52].

The median estimated total daily caffeine in the current study (146.73 mg·day^−1^) was greater than the median but less than the mean caffeine intake reported in the NZ ANS08/09 (123 mg·day^−1^ and 196 mg·day^−1^, respectively) [17]. This was also less than 1369.92 mg/week (~195 mg·day^−1^) of caffeine consumed among psychology students in the US, which was most commonly sourced from coffee and espresso/lattes [53]. In a later study of US college students, caffeine intake was lower than both the current study and the earlier US study [53], however, coffee remained the main source of caffeine intake in male (120 mg·day^−1^) and female (111 mg·day^−1^) caffeine consumers [55]. An earlier UK-based study [56] found that coffee, tea and soft drinks were the greatest contributors to mean daily caffeine intakes for students (141.92 mg, 51.86 mg and 11.90 mg, respectively). However, the UK study [56] did not include all of the caffeine-containing products available on the market today (i.e., energy drinks/shots, RTD and sports supplements were not included). In summary, although studies have used different methods to assess caffeine consumption, it is evident that coffee is the largest contributor to caffeine intake in tertiary students in several countries.

Energy drinks were consumed by 40.4% of tertiary students; this is higher than the 3.1% estimated in a sub-analysis of the NZ ANS08/09 (*n* = 138/4452) [17]. A rise in energy drink consumption since 2008/2009 [17] is expected, as worldwide energy drink consumption reportedly doubled between 2006 and 2012 [57] and previous research has suggested that energy drink consumption is generally higher in tertiary students than in the general population [53,58]. Our results indicate that the frequency of energy drink consumption differs between tertiary students (the majority of consumers only consumed energy drinks 1–3 times per month) and the general population (79.9% of energy drink consumers consume one serving per day) [17].

The median estimated total daily caffeine consumption of 2.25 mg·kgbw^−1^·day^−1^ is below the adverse effect level (3 mg·kgbw^−1^·day^−1^) [17], but is greater than the median caffeine intake from the NZ ANS08/09 (1.6 mg·kgbw^−1^·day^−1^) [17]. In the present study, consumption of caffeine tablets, coffee, tea and caffeinated RTD were associated with the proportion of participants who exceeded the adverse effect level. Over one-third (34.4%) of participants consumed caffeine in excess of the adverse effect level, similar to but greater than the NZ ANS08/09 where 30% of NZ adults (including pregnant women, for whom the adverse effect level was 200 mg·day^−1^) exceeded the adverse effect level. However, the NZ ANS08/09 did not include caffeinated RTD or caffeine tablets, which may explain why the median estimated intake was lower and fewer respondents (%) exceeded the adverse effect level than in the present study.

Furthermore, approximately 15% of participants in this study and almost 20% reported from a convenience sample of 2379 NZ adults [59] consumed caffeine in excess of the ‘safe limit’ (400 mg·day^−1^). Smokers had a higher total daily caffeine consumption than non-smokers, and were 3.58 times more likely to exceed the ‘safe limit’ of caffeine consumption, however only 14.8% of participants were smokers, compared to 16.3% of NZ adults in 2015/16 [60], suggesting that this educated group, aged ≥ 16 years old, are less likely to smoke than the general adult population. Alcoholic RTD and coffee consumers were 2.26 times and 16.29 times more likely, respectively, to exceed the ‘safe limit’ of caffeine intake than non-consumers of these products. The latter is not surprising given that coffee was the greatest contributor to total caffeine intake in this study. However, the former (alcoholic RTD) may be related to drinking behaviours, such as “drinking to get drunk” and “to have fun”, and opting for palatable products with higher alcohol content [61,62], resulting in a larger volume of the alcoholic RTD and (therefore) caffeine consumed. Future NZ studies should aim to quantify caffeine consumed, from all sources, when co-ingested with alcohol, to better understand this risk-taking behaviour.

Although there was no difference in the proportion of participants exceeding 400 mg·day^−1^, or in total estimated daily caffeine consumption according to gender, relative daily consumption by weight was significantly higher in females than males. Metabolism of caffeine in females is 20–30% faster than that of males [20], therefore females may be able to consume relatively higher amounts of caffeine without experiencing more adverse symptoms than males. Additionally, as caffeine is not distributed in body fat the same way as it is in lean mass, the same relative intake in a healthy weight individual will not have the same effect in overweight people (i.e., an overweight individual experiencing greater effects) [63]. Fulgoni [63] suggests that in overweight individuals, caffeine consumption limit guidelines should be adjusted according to ideal body weight (i.e., if BMI was 25 kg/m^2^). However, BMI is not always an accurate measure of excess adipose tissue and may indicate high muscle mass in some cases [64].

The 15–20% of NZ adults exceeding the ‘safe limit’, and the related behaviours (smoking and co-consumption with alcohol), paired with the 30–34% of the general public and study participants who consumed caffeine in excess of the adverse effect level, suggests a potential public health issue, as caffeine consumption above these levels may lead to substance-related harm (such as caffeine intoxication) and increases in anxiety, respectively. The majority (84.7%) of caffeine consumers in this study reported experiencing at least one symptom post caffeine consumption. Similarly, 85% of adult respondents (≥18-years-old) in a NZ-based study reported at least one caffeine-related harm event (e.g., dehydration, feeling dependent, insomnia, irritability, headache) in the past year [59]. In the present study, the caffeine sources for which the highest proportion of participants reported symptoms post consumption were energy drinks (77.3%) and coffee (76.9%). These were also the two caffeine sources which had the highest levels of self-reported dependence (32.8% and 59.3% of consumers respectively). These results may be related to the relatively high caffeine content of these products or the fact that they are commonly known to contain high caffeine [52] and therefore the participants expected these symptoms.

Nearly half of the respondents (*n* = 152, 47.9%) reported suffering withdrawal symptoms after stopping consumption of caffeine. Of these participants nearly one third (*n* = 42, 27.6%) experienced more than one withdrawal symptom, and nearly half (*n* = 68, 44.7%) reported that their symptoms had an impact on their social life or work life or caused some kind of distress. In comparison, nearly one-third (30%) of participants in another NZ-based study by Booth et al. met DSM-5 (Diagnostic and Statistical Manual of Mental Disorders 5th edition) criteria for caffeine withdrawal [59]. The DSM-5 criteria for the diagnosis of caffeine withdrawal requires the fulfilment of four criteria including: (a) prolonged daily use of caffeine; (b) abrupt cessation/reduction in caffeine use resulting in three or more specific symptoms within 24 h; (c) that the symptoms from (b) cause clinically significant distress or impairment; and (d) the signs/symptoms are not the result of something else [65]. Thus, the discrepancy between the present study, where withdrawal symptoms were reported by nearly half of the respondents (47.9%), and the 30% of participants reported by Booth et al. [59] may be due to the differing criteria, i.e., the experience of ≥1 withdrawal symptom in the present study versus achieving four criterion (A–D) for the diagnosis of caffeine withdrawal in the latter [59] study.

Retrospective data collection relies on accuracy of the participants’ memory, which can lead to inaccuracy, however for any data collection involving questionnaires, some level of recall error is unavoidable [66]. While efforts were made to obtain accurate and current data on the caffeine content of a wide range of products, it is inevitable there will be some error in the consumption estimates due to difficulty ascertaining the exact caffeine content and the consumption patterns of the wide range of products available. For example, the caffeine content(s) of the two greatest contributors to caffeine intake (coffee, tea) and the three most universally consumed caffeine-containing products (coffee, tea, chocolate) in this study are not regulated or required to be reported. These products naturally contain caffeine and the caffeine content may differ due to growing conditions, species or variety (Robusta coffee beans generally contains twice the caffeine of the Arabica variety), composition (the higher percentage of cocoa bean solids in dark chocolate gives it a higher caffeine content than milk chocolate) and processing and brewing techniques [8,9,10].

The extensive inclusion of caffeinated products is a strength of this study. This is important in determining the contribution of different caffeinated products to total caffeine consumption to identify which of these products may require special attention with regards to ameliorating caffeine-related risk. However, to reduce time taken and participant burden, CaffCo only contains the most common variants of each product group (e.g., main types of coffee or tea) which limits CaffCo’s ability to quantify the caffeine intake levels of individuals. To fully quantify caffeine intake in individuals, a separate caffeine FFQ would need to be developed that included all caffeinated products available on the NZ market.

A nationwide study to assess caffeine intake among a representative sample of New Zealanders using the CaffCo questionnaire is warranted. NZ is an ethnically diverse nation and understanding caffeine intake among indigenous Māori using culturally appropriate recruitment methods would strengthen our enquiry.

## 5. Conclusions

Approximately 15% of the sample participants reported consuming caffeine in excess of the daily ‘safe limit’ and 34% above the adverse effect level, which suggests a potential public health issue. Coffee, followed by tea and energy drinks, were the biggest contributors to daily caffeine intake in NZ tertiary students. Factors which put tertiary students at a higher risk of exceeding this ‘safe limit’ were smoking, consumption of coffee and consumption of caffeinated RTD. As the benefits and risks are dose dependent, the public health consequences of caffeine consumption can only be determined once data is available on the amount of caffeine currently being consumed by New Zealanders.

The present study provides useful data for multiple stakeholders (e.g., the scientific community, public health professionals, regulatory agencies, consumers, retailers and the food industry) in regards to the caffeine consumption of NZ tertiary students, and provides the basis for developing strategies (e.g., improved labelling, consumer education, additional regulations etc.) in order to reduce or ameliorate caffeine-related risk in this population group.

## Figures and Tables

**Table 1:** Socio-demographic and anthropometric characteristics, sources of caffeine.

Variable	Participants, *n* = 317 *n*(%)/Median [IQR]
Sex	
Male	148 (46.7)
Female	169 (53.3)
Age group	
16–18 years	51 (16)
19–30 years	236 (74.4)
31–50 years	25 (7.9)
51+ years	5 (1.6)
Ethnicity ^1^	
NZ European	150 (47.3)
Other European	56 (17.7)
Māori	17 (5.4)
Asian	128 (40.4)
Pacific Peoples	22 (6.9)
Middle Eastern/Latin American/African	16 (5.0)
Living situation	
Living alone	23 (7.4)
Living with family	174 (54.9)
Flatting with others	108 (34.1)
Halls of residence	7 (2.2)
Living with partner	5 (1.6)
Employment status	
No paid employment	211 (66.6)
Part-time employment	102 (32.2)
Full-time employment	4 (1.3)
Body mass index, BMI (kg/m^2^) ^2^	
Total	22.9 [20.8, 25.1]
Male	23.4 [21.0, 26.1]
Female	22.3 [20.3, 24.2]

^1^ Percentage exceeds 100 as participants were able to select more than one ethnicity. ^2^ *n* = 263 participants provided body weight and height data.

**Table 2:** Estimated daily caffeine consumption from caffeine sources by gender.

Caffeine Source	Male, mg·day^−1^	Female, mg·day^−1^	Mann-Whitney (U)	*p*
Tea	26.43	26.53	6207.5	0.996
Coffee	100.00	108.76	6825.0	0.496
Chocolate	8.91	8.88	8112.0	0.864
Kola drinks	15.31	9.94	2046.0	<0.001
Energy drinks	32.20	11.54	1191.0	<0.001
Caffeinated RTD	6.10	3.00	275.0	0.022
Supplements ^1^	90.52	19.34	31.0	0.176
Caffeine tablets	3.30	6.70	8.5	0.282

RTD: ready to drink alcoholic beverage. ^1^ Caffeine containing sports supplements.

**Table 3:** Proportion of participants who reported dependence on caffeine sources.

Caffeine Source	Proportion of Participants Who Are Dependent (%) ^1^
Tea (*n* = 227)	24.8
Coffee (*n* = 242)	59.3
Chocolate (*n* = 259)	19.8
Kola drinks (*n* = 156)	8.3
Energy drinks (*n* = 128)	32.8
Caffeinated RTD (*n* = 58)	1.7
Caffeine-containing sports supplements (*n* = 21)	19.0
Caffeine tablets (*n* = 11)	18.2

RTD: ready to drink alcoholic beverage. ^1^ Of those who consume the product.

## Data Availability

The data presented in this study are available on request from the corresponding author.
